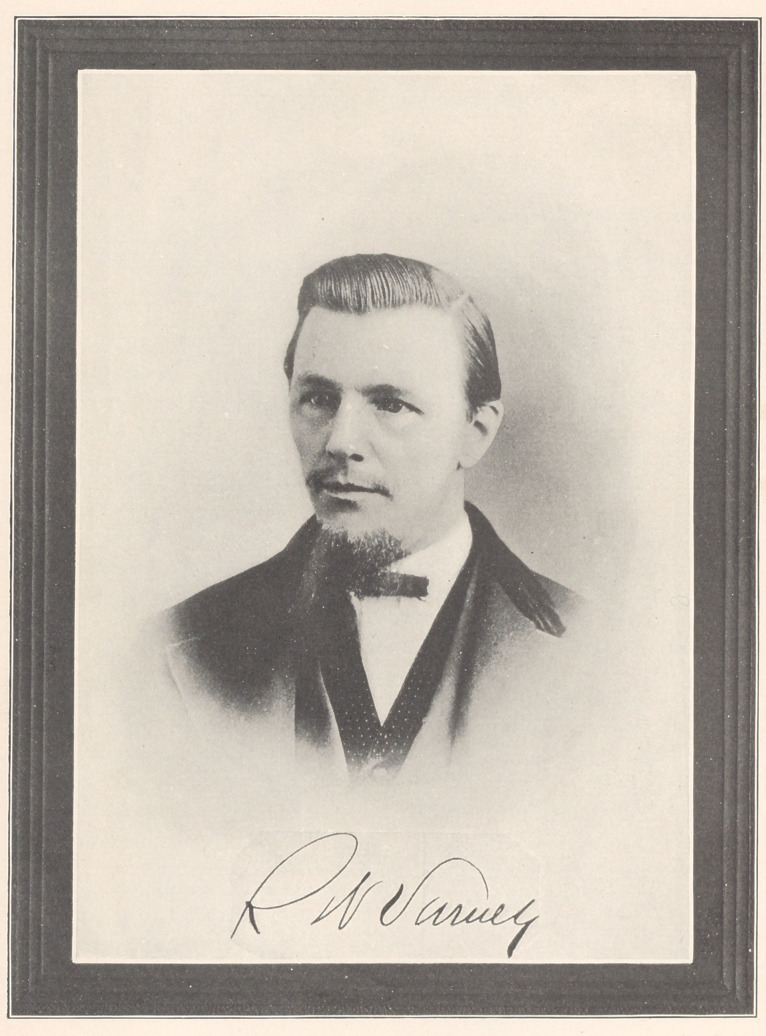# Royal William Varney, M.D.

**Published:** 1902-04

**Authors:** 


### Biographical Sketch.

### ROYAL WILLIAM VARNEY, M.D.

Royal William Varney was born at Independence, Ohio, October 8, 1839, and early in life removed to Newburg, Ohio, where he received a common-school education, and later became a pupil of Dr. William H. Atkinson. About 1861 he accompanied his preceptor to New York and completed his term of pupilage. Subsequently returning to Ohio, he graduated in medicine at one of the schools of that State in 1863.

Dr. Varney soon afterwards accepted a commission as assistant surgeon in the Thirty-first Ohio Volunteer Infantry, and went with Sherman on his march to the sea. Never a very strong man physically, it is probable that the hardships and exposure of army life contributed to his untimely death. At the close of the war he became associated with Dr. George E. Hawes in New York City. He was afterwards located in a house of his own in West Thirty-sixth Street. He died at Savannah, Georgia, April 12, 1872, in the thirty-second year of his age.

Dr. Varney received the degree of Master of Dental Surgery from the Dental Society of the State of New York, June 29, 1871. He was a member of the Society of Dental Surgeons of the City of New York, of the First District Dental Society, and of the American Microscopical Society.

To his initiative was due the system of public clinics which prevailed for many years in the First District Society, and which has spread far and wide, to the great benefit of the practice of dentistry.

Not much of a talker, he was one of the men "who *do* things," content to write his autobiography in the mouths of his patients. While skilful in the highest degree, resourceful in overcoming difficulties, and having full confidence in himself, yet he who might have applied to himself the words of Shakespeare, "This hand was made to handle naught but gold," was extremely modest and never boasted.

Not merely an expert manipulator,—a dental virtuoso,—he was thoughtful and studious, and sought the causes of the phenomena he observed, working much with the microscope.

As a man he was inflexible in his integrity, possessing a soul of honor, a gentle and loving disposition, and some of the older dentists to-day remember him as a warm and true friend.

In response to inquiries, the following interesting letter was received from Dr. S. G. Perry:

"New York, February 25, 1902.

"In presenting a biographical sketch of Dr. Varney your first concern is for facts. Opinions are only side-lights that help illumine your subject. In response to your letter I can only say that I am not able to give you many facts, but I will venture the opinion *that he was one of the most remarkable men the profession has produced.*

"He was a natural mechanic, and to the art of finger-craft he added great force of will. He dared to attempt to do perfect work at a time in our profession when few men believed the public would submit to the annoyance, the pain, and the expense necessary to produce it. He was probably one of the first men in the profession to charge by the hour for his services.

"He died at thirty-two, and yet he left work that must remain a model for all time. His method was most simple. He opened his cavities freely, to admit the use of nearly straight instruments, annealed his gold himself to different degrees of cohesiveness, and condensed it for the most part with the lead mallet in his own hands.

"His set of pluggers tell the story of his method. His sense of form was well developed, and he contoured his fillings beautifully.

"I have no objection to your using the following extract from the Transactions of the Odontological Society. I do not take back a word of it. It is as true to-day as when it appeared in 1887.

"'I think it is safe to say that Dr. Varney reached high-water mark in the art of filling teeth. No one who came before, and no one, to my knowledge, who came after, excelled him in saving teeth. I have seen a great deal of his work (much of it now of twenty years' standing), and I have never seen, nor heard of, a filling of his that was not contoured. He said to me once that his ambition was, if cut short in his work at any moment, the last filling he put in should be his best,—the one he would be content to be judged by.'

"So far as I know, he wrote but two short papers, one on 'Root-

Filling,' the other having been published in the *Dental Cosmos* for April, 1871. He was too modest to allow his name to appear in connection with it. It was on the subject of the preparation of cavities, and it was so concise, and so to the point, that I think it would be well to reprint it at this time. It is only just to his memory, for as it stands it is unsigned, and there are now few living that could testify to its authorship. The *italics* are his own, and give an indication of his force of character.

"It is peculiarly gratifying to me that some one has been willing to take the trouble to revive his memory.

"Yours most truly,

"S. G. PERRY."

"PREPARATION OF CAVITIES.

[Read before the New York Odontological Society, November 15, 1870, by Dr. R. W. Varney.]

"Thoroughly cleanse the teeth, if needed, as is most likely the case. Medication, if used at all, to allay pain in excavating, ought to be made use of early. The common method of partially preparing, and *then* applying remedies in *hopes* of lessening the suffering, I have no patience with. Having little or no faith in local applications other than a *keen edge vigorously applied;* scarcely anything else is used at my hands. Wash out the cavity with warm water, dry, and look at it. If the pulp is not then exposed, it is an inexcusable blunder, unworthy a professional man, if it becomes so during the operation. Cut away the margin as far as required, for strength or otherwise. Form the walls, by cutting from *towards* the pulp *outward,*—never by cutting from without inward; *towards* the pulp, if the dentine is much softened. Leave the deeper portion of the cavity untouched, if need be, rather than expose or uncover a pulp, for it can never be protected so nicely again, except by secondary dentine. The practice of purposely exposing pulps that are only protected by a thin portion of softened dentine, for the purpose of capping with oxychloride of zinc, as advocated by some of the teachers, I most emphatically object to. Never remove darkened dentine simply because it *is* darkened, unless situated so that it is likely to show after completion of the operation. I would never wish to have a grinding surface, buccal, labial, or lingual surface cavity undercut, but would prefer to have the walls parallel. Approximal grinding ones should be somewhat undercut, when considered from their approximal aspect, as more retaining power is required in such cases. Have the floor, or part to be built from, as near a plane surface as is consistent with safety, and the lateral walls rising perpendicularly from it. I do not drill retaining-pits, but prefer to hold the first few pieces till self-sustaining."

I am particularly indebted to Dr. J. S. Latimer, of New York, for the facts in regard to the life of one who will always occupy a unique position in the history of the dental profession.

CHARLES MCMANUS.

## Figures and Tables

**Figure f1:**